# No Effect of Dose Adjustment to the *CYP2D6* Genotype in Patients With Severe Mental Illness

**DOI:** 10.3389/fpsyt.2018.00349

**Published:** 2018-08-07

**Authors:** Anne B. Koopmans, David J. Vinkers, Igmar T. Poulina, Petra J. A. Gelan, Ron H. N. van Schaik, Hans W. Hoek, Peter N. van Harten

**Affiliations:** ^1^Parnassia Academy, Parnassia Psychiatric Institute, The Hague, Netherlands; ^2^School of Mental Health and Neuroscience, Maastricht University, Maastricht, Netherlands; ^3^Klinika Capriles Willemstad, Curaçao; ^4^Department of Clinical Chemistry, Erasmus University Medical Center, Rotterdam, Netherlands; ^5^Department of Psychiatry, University Medical Center Groningen, Groningen, Netherlands; ^6^Department of Epidemiology, Mailman School of Public Health, Columbia University, New York, NY, United States; ^7^Innova, Psychiatric Centre GGz Centraal, Amersfoort, Netherlands

**Keywords:** CYP2D6, severe mental illness (SMI), guidelines, dose adjustment, genotyping, psychopharmacology, personalized medicine

## Abstract

**Background:** The CYP2D6 enzyme is involved in the metabolism of numerous psychopharmacological drugs. Guidelines recommend how to adjust the dose of medication based on the *CYP2D6* genotype.

**Aims:** To evaluate the effect of dose adjustment to the *CYP2D6* genotype and phenotype, in patients with severe mental illness (SMI) already receiving psychopharmacological treatment.

**Methods:** A total of 269 psychiatric patients (on the island Curaçao) receiving antipsychotic treatment were genotyped for *CYP2D6*. Of these, 45 patients were included for dose adjustment according to the clinical guideline of the Royal Dutch Association for the Advancement of Pharmacy, i.e., 17 CYP2D6 poor metabolizers, 26 intermediate metabolizers, and 2 ultrarapid metabolizers. These 45 patients were matched for age, gender, and type of medication with a control group of 41 patients who were CYP2D6 extensive metabolizers (i.e., with a normal CYP2D6 function). At baseline and at 4 months after dose adjustment, subjective experience, psychopathology, extrapyramidal side-effects, quality of life, and global functioning were assessed in these two groups.

**Results:** At baseline, there were no differences between the groups regarding the prescribed dosage of antipsychotics, the number of side-effects, psychiatric symptoms, global functioning, or quality of life. After dose adjustment, no significant improvement in these parameters was reported.

**Conclusion:** In psychiatric patients with SMI already receiving antipsychotic treatment, dose adjustment to the *CYP2D6* genotype or phenotype according to the guidelines showed no beneficial effect. This suggests that dose adjustment guidelines are currently not applicable for patients already using antipsychotics.

ClinicalTrials.gov: Cost-effectiveness of CYP2D6 and CYP2C19 Genotyping in Psychiatric Patients in Curacao; Identifier: NCT02713672; https://clinicaltrials.gov/ct2/show/NCT02713672?term=CYP2D6&rank=5

## Introduction

The cytochrome P450 isozymes, in particular CYP2D6, is responsible for the biotransformation of many psychopharmacological drugs (1, 2). Substrates of CYP2D6 include first generation antipsychotics, selective serotonin receptor inhibitors and tricyclic antidepressants^1^. Based on genetic variation, patients can be divided into poor metabolizers (PM), intermediate metabolizers (IM), extensive metabolizers (EM), and ultrarapid metabolizers (UM). The recommended dosages of psychopharmacological medication that are metabolized by this enzyme are based on the metabolism of the most common genotype, i.e., the EM type (i.e., a normal CYP2D6 function). However, because the plasma level of a drug is related to the genotype, the same dosage will probably lead to a higher plasma level in PMs and IMs, as compared to EMs, and to a lower plasma level in UMs as compared to EMs. The plasma level is often related to the effectiveness of the drug and the risk of dose-related side-effects (3–7). Also, when physicians prescribe a drug metabolized by CYP2D6 without taking into account the genotype, the hospital stay is longer (and the costs higher) in patients with a PM and UM profile (8).

Clinical guidelines recommend dose adjustment according to the *CYP2D6* genotype (9–11). However, the current guidelines do not differentiate between patients that start vs. those that are already receiving psychopharmacological treatment. Patients with severe mental illness (SMI) are especially known to suffer from problems with adverse drug reactions, lack of medication effect, and new models of care are warranted (12–16). In a study in patients with SMI, more adverse drug events and higher costs were found in the extreme metabolizer groups (17). In a cost analysis study it was found that genotyping in patients with schizophrenia could lead to lower treatment costs (18).

Genotyping in patients with SMI could potentially individualize treatment, reduce side-effects in slower metabolizers and increase treatment effects in rapid metabolizers. Until now, it remains unclear whether routine *CYP2D6* genotyping is efficacious in patients with SMI already undergoing psychopharmacological treatment and evidence of clinical utility of *CYP2D6* genotyping in patients being prescribed antipsychotics is lacking (19–21). We hypothesized that dose adjustment of antipsychotics to the *CYP2D6* genotype and phenotype would be beneficial regarding side-effects, psychiatric symptoms, quality of life, and/or global functioning. The aim of the present study was to evaluate the effect of dose adjustment to the *CYP2D6* genotype and phenotype, in patients with SMI already receiving psychopharmacological treatment. The dose adjustment group consisted of patients with a PM, IM, or UM profile using antipsychotics metabolized by CYP2D6, whereas the control group consisted of patients with an EM geno-/phenotype. The effect of dose adjustment of the antipsychotics on psychopathological symptoms, side-effects, and well-being was evaluated.

## Methods

### Patients

This study was performed on the Caribbean island, Curaçao: this is one of the western Leeward Antilles in the Caribbean with about 160,000 inhabitants^2^. Patients were recruited via the *Klinika Capriles* (the psychiatric hospital on the island), the psychiatric ward of the local prison (FOBA), and the psychiatric outpatient clinic (*Psychiaters Maatschap Antillen*).

After being informed about the study procedures, all patients signed written informed consent. Inclusion criteria were: Antillean ethnicity (defined in line with the concepts used by the Central Office of Statistics in the Netherlands, as birth on the former Netherlands Antilles and birth of at least one parent on the former Netherlands Antilles); age ≥18 years; use of an antipsychotic or antidepressant drug; able and written informed consent. All participants in both groups received a token for 25 Netherlands Antillean Guilder (about US $13) if they completed the study.

All DNA samples were genotyped July 2012 for *CYP2D6* ^*^1, ^*^2, ^*^3, ^*^4, ^*^5, ^*^6, ^*^7, ^*^8, ^*^9, ^*^10, ^*^17, ^*^29, ^*^41 and gene duplication in the Erasmus Medical Center (Rotterdam, the Netherlands) and grouped according to the predicted phenotype for *CYP2D6* as described earlier (22).

Diagnoses, demographic information and information on psychiatric and somatic medication was derived from the electronic patient file. The following psychiatric drugs were considered to have a major dependence on the CYP2D6 enzyme for their elimination: amitriptyline, aripiprazole, atomoxetine, clomipramine, imipramine, haloperidol, nortriptyline, paroxetine, pimozide, risperidone, venlafaxine, and zuclopenthixol^1^ (9, 10).

Based on the *CYP2D6* genotype or phenotype, patients were selected who were recommended a dose adjustment of their psychopharmacological medication according to the guideline of the Royal Dutch Association for the Advancement of Pharmacy (updated until July 2013). In this guideline, recommendations were developed for 53 drugs based on a systematic review of the literature. In CYP2D6 PM, IM, or UM patients, using medication metabolized by CYP2D6, it is advised to switch to a drug that is not metabolized by CYP2D6. An alternative is to adjust the dosage with dose reductions of respectively 25–50% of the original dose in IMs and PMs (9, 10).

To increase the power of the present study, patients who were PM or IM based on inhibiting medication were also included in the dose adjustment group (23).

Patients using strong CYP2D6 inhibitors (bupropion, cinacalcet, fluoxetine, paroxetine, quinidine) according to Flockhart's interaction table, were classified as being PM^1^ (23, 24).

The selected patients were matched for age, gender and type of medication with a control group of patients who were CYP2D6 extensive metabolizers.

All prescribed antipsychotics were calculated to a “defined daily dose” (DDD) as reported by the World Health Organization (WHO) (25). This is a unit of measurement and defined as the assumed average maintenance dose per day for a drug used for its main indication in adults. The total equivalent of the DDD was calculated for every patient. The study protocol was approved by the Institutional Review Board of Maastricht University (the Netherlands) and ethical approval to collect DNA samples was received according to local policies by the Institutional Review Board of the Klinika Capriles (Curaçao).

The study was registered in an international trial registry at http://www.clinicaltrials.gov (NCT02713672). All procedures were in accordance with the ethical standards of the Declaration of Helsinki 1975 (as revised in 1983).

### Assessments

Each patient underwent a thorough assessment of psychopathology, subjective experience, extrapyramidal symptoms, quality of life, global functioning, and metabolic parameters at baseline (T0) (November–December 2014) and at 4 months after dose adjustment (T1) (April–June 2015). Information about drug and alcohol use was registered.

The severity of the patient's psychopathology was assessed with the Brief Psychiatric Rating Scale (BPRS) (26). Extrapyramidal symptoms were assessed with the St. Hans Rating Scale (SHRS) (27). Akathisia was measured with the Barnes Rating Scale for drug-induced akathisia (BARS) (28, 29). Quality of life was assessed with the EQol 5-D (EQ 5-D) (30). Global functioning was assessed with the WHO Disability Assessment Schedule 2.0 36-item proxy-administered version (WHODAS 2.0) and was administered with a personal caregiver (31, 32); scores were recalculated to a standardized score. In all above-mentioned scales, higher scores indicate more severe symptoms. Subjective experience was measured with the Subjective Well-Being Under Neuroleptics Scale (SWN-20) (33). Scores were recalculated, with higher scores indicating a higher level of well-being. For patients who were unable to read Dutch, the questions were translated into Papiamento (a local language). The investigator, a resident in psychiatry, was trained by professionals in scoring the SHRS, BARS and the WHODAS 2.0. Patients receiving depot medication were measured the same number of days after administration of the depot at T0 and at T1.

Secondary outcome measurements were metabolic parameters (blood pressure, body mass index (BMI), waist size, cholesterol, HDL, LDL, triglycerides, glucose, HbA1Cl, and prolactin). Also in 31 of the 60 patients receiving antipsychotics metabolized by CYP2D6 (dose adjustment group *n* = 22, control group *n* = 9), the plasma levels of antipsychotics were measured.

### Procedures

After baseline measurements, another psychiatrist in training prescribed the dose adjustments. A standard procedure for dose adjustments was followed. Lowering the dose was done in steps according to a local protocol^3^ (34). Tranquilizing medication with inhibitory activity was replaced by benzodiazepines. By removing the inhibiting medication, no further dose reduction was necessary in these patients. Complex cases were discussed with the research team during a regular meeting and individual dose adjustment plans were made.

### Statistical analysis

Analyses were performed with IBM SPSS statistics (version 22). Differences between groups were tested with a Chi-square test for dichotomous variables and an independent *t*-test for continuous variables. ANOVA was used to compare means between the geno-/phenotypes. Non-parametric tests were used for variables not normally distributed (i.e., SHRS, BARS, EQ 5-D). Differences at T1 that were present at T0 were corrected for in an ANCOVA model. The relation between geno-/pheno and DDD was investigated with Kendall's tau. All tests were two-tailed. The significance level was set at *p* < 0.05. After Bonferroni correction, the significance level used was *p* < 0.005 (0.05/11).

To analyse all aspects of deterioration, psychiatric well-being was evaluated on (one of) three scored items: i.e., deterioration was defined as a specific report by caregivers or a physician, or a >5-point increase on the BPRS, or on the WHODAS 2.0.

A *post-hoc* power analysis showed that a 25% reduction of the psychiatric symptoms, or a 30% reduction of the symptoms measured by WHODAS 2.0, or a 75% reduction of the Parkinson symptoms, results in a power of 80% at a significance level of 0.05.

## Results

### Baseline characteristics

The study population consisted of 269 long-term psychiatric patients, of which 94% was diagnosed with a psychotic disorder. Other diagnoses were major depressive disorder, bipolar disorder, substance abuse and intellectual disability. No regular drug or alcohol use was reported in any of the questionnaires. A quarter of the patients was outpatient. The majority (92%) was admitted in a long-term treatment facility or institutional housing at the start of genotyping and had a long treatment history. Every patient was genotyped 2.5 years before the start of the intervention, and for at least this period in treatment. Patients were stable on antipsychotic treatment for at least 2.5 years, three patients switched once from antipsychotic during this treatment period.

Genotyping succeeded in 231 participants. Failure of genotyping was due to the low quality DNA obtained from the buccal swabs. Frequencies of PMs, IMs, and UMs were similar to those found in an Antillean population without psychiatric disease (22); Caucasian populations (22); and in patients using antipsychotic medication not metabolized by CYP2D6.

**Figure 1:**
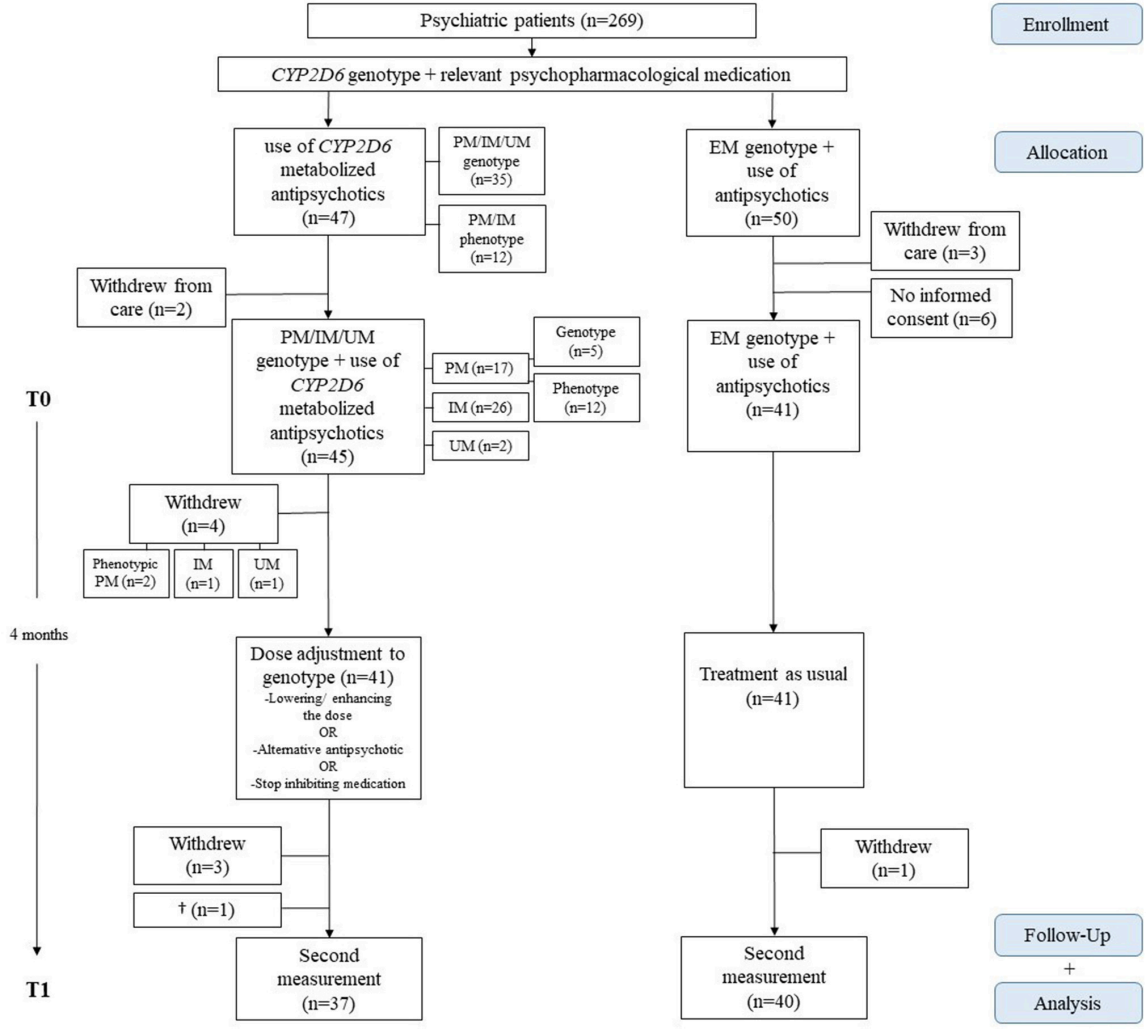
Study procedure and inclusion of participants. PM, poor metabolizer; IM, intermediate metabolizer; UM, ultrarapid metabolizer; EM, extensive metabolizer.

In total 111 out of 269 patients were using medication metabolized by CYP2D6. In total, 153 patients were prescribed two types of antipsychotics and 24 patients used antidepressant medication. Because the admitted patients received their medication from the nurses and two third of the patients were using depot medication we could account for medication adherence in 90% of the included patients. Figure 1 presents an overview of the patient selection and study procedure. The medication in the dose adjustment group was haloperidol (*n* = 15), risperidone (*n* = 21), and zuclopenthixol (*n* = 9).

Out of 41 patients, 16 patients received a dose reduction. The mean dose reduction was 0.60 DDD, (1.4 DDD in PMs, 0.54 DDD in IMs) which equals a reduction from 5 to 2 mg risperidone. Other patients stopped with their inhibiting medication or received alternative antipsychotic medication. Table 1 presents the patient characteristics and the outcome of measurements at T0 and T1.

**Table 1:** Clinical characteristics, baseline, and delta scores of the dose adjustment and control group.

	**T0 dose adjustment group *n* = 45 Mean (*SD*)**	**T0 control group *n* = 41 Mean (*SD*)**	*p*-value^*^	**T1-T0 dose adjustment group *n* = 37 Mean (*SD*)**	**T1-T0 control group *n* = 40 Mean (*SD*)**	*p*-value^*^
Male (*n*)	30	25	0.58			
Female (*n*)	15	16	0.58			
Age (years)	52.4 (12.0)	50.3 (10.8)	0.15			
Depot medication	30	27	0.59			
Outpatient	14	9	0.34			
Defined daily dose	1.65	1.92	0.17			
BPRS: 24 items (1–7)	1.79 (0.51)	1.66 (0.43)	0.26	−0.26 (0.26)	−0.17 (0.27)	0.15
SWN-20: 20 items (1–6)	4.59 (0.95)	4.45 (1.02)	0.59	0.29 (0.66)	−0.06 (0.57)	0.04
WHODAS 2.0: 32 items (1–5) (standardized total score)	32.06 (16.28)	30.40 (16.89)	0.69	5.47 (17.50)	2.93 (10.63)	0.52
EQ 5-D: 5 items (1–3)	1.30 (0.31)	1.31 (0.38)	0.78	−0.07 (0.37)	−0.05 (0.24)	0.74
Dyskinesia SHRS: 18 items: (0–6)	0.61 (0.70)	0.78 (0.87)	0.58	0.19 (0.55)	0.045 (0.57)	0.33
Parkinsonism SHRS: 10 items (0–6)	0.97 (1.12)	1.00 (1.33)	0.70	0.47 (0.76)	0.04 (0.72)	0.05
Dystonia SHRS: 2 items (0–6)	0.06 (0.34)	0.27 (1.05)	0.36	0 (0.51)	−0.20 (1.00)	0.70
BARS: 3 items (0–3)	0.10 (0.40)	0.37 (0.73)	0.01	0.13 (0.54)	−0.26 (0.70)	0.25^**^
Blood pressure (mmHg)	124/81 (14/10)	125/79 (17/11)	0.60	1.50 (15.48) /−0.76 (9.72)	−1.89 (15.29) /−0.76 (9.72)	0.37
BMI	26.6 (6.4)	27.3 (6.7)	0.66	−0.05 (1.51)	−0.64 (1.45)	0.12
Cholesterol (mg/dl)	159.8 (37.7)	160.0 (34.4)	0.99	8.8 (22.6)	6.4 (13.6)	0.63
HDL (mg/dl)	45.4 (15.1)	42.4 (10.7)	0.34	3.5 (6.5)	1.2 (6.3)	0.19
LDL (mg/dl)	92.7 (33.8)	92.1 (31.3)	0.94	6.4 (19.8)	8.1 (20.6)	0.76
Triglyceride (mg/dl)	104.5 (41.4)	111.6 (56.7)	0.57	−5.7 (28.4)	−6.6 (38.6)	0.93
Prolactin (ng/ml)	37.3 (62.1)	20.2 (14.4)	0.22	−13.0 (37.5)	2.6 (10.0)	0.11
Fasting glucose (mg/dl)	112.0 (25.1)	103.2 (32.0)	0.24	−4.1 (14.8)	−4.2 (17.4)	0.98
HbA1c %	4.5 (1.5)	5.4 (1.5)	0.33	−0.1 (0.0)	−0.08 (0.43)	0.95

* *Significance level after Bonferroni correction p < 0.005*.

** *ANCOVA test corrected for differences at T0 that were present at T1; BPRS, Brief Psychiatric Rating Scale; SWN-20, Subjective Well-being Under Neuroleptics; WHODAS, World Health Organization Disability Assessment Schedule; SHRS, St. Hans Rating Scale; BARS, Barnes Rating Scale for drug-induced akathisia*.

Four months after dose adjustment (T1), 81 (94%) patients were assessed for the follow-up measurements; one patient had died of cancer, two patients had withdrawn from psychiatric care, and two patients did not want to participate a second time.

### Baseline (T0)

**Figure 2:**
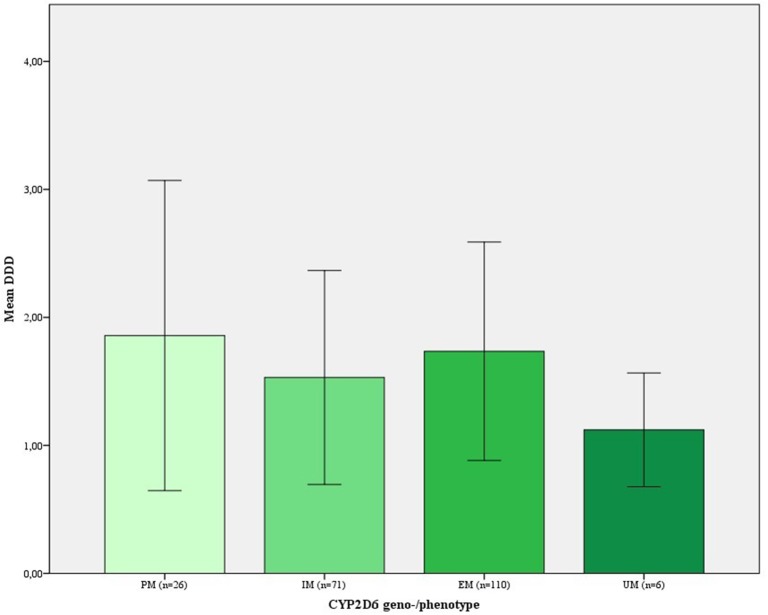
Prescribed dose of antipsychotics in DDD per CYP2D6 geno-/phenotype group at baseline. DDD, defined daily dose; PM, poor metabolizer; IM, intermediate metabolizer; UM, ultrarapid metabolizer; Error Bars, ±1 SD.

At baseline, no differences were found between the geno-/phenotype and the mean prescribed dose of antipsychotics as shown in Figure 2. In the dose adjustment group the mean DDD was 1.65 (SD 0.83) and in the control group it was 1.92 (SD 0.97) (Ns). PMs, IMs and UMs were prescribed the same amounts of psychopharmacological medication as the EMs.

Second, we found no difference in dose-dependent adverse drug reactions between the normal and extreme metabolizers. Movement disorders were equally distributed in both groups. There were no differences in metabolic parameters.

Third, no differences were found between the normal and extreme metabolizers for psychiatric symptoms, subjective well-being and quality of life. There were no differences in psychiatric symptoms as measured by subscales of the BPRS.

Because only 13 of the patients worked, the four items of the WHODAS 2.0 concerning work were omitted. Patients with a PM genotype or phenotype scored higher on the WHODAS 2.0 than the IMs (PM 41.5, IM 26.6, EM 29.1, UM 34.0) (*p* = 0.007); however, this difference was not significant after Bonferroni correction (*p* = 0.005).

Fourth, no significant differences were found between the dose adjustment and the control group in mean therapeutic drug plasma levels of antipsychotics metabolized by CYP2D6 (analyzed with the Mann-Whitney *U*-test). The mean therapeutic plasma levels at T0 of respectively the dose adjustment and the control group were for haloperidol 0.0015 mg/l (*SD* 0.0013) (*n* = 4) and 0.0023 mg/l (*SD* 0.0006) (*n* = 3) (*p* = 0.35); risperidone 0.0173 mg/l (*SD* 0.0164) (*n* = 9) and 0.0073 mg/l (*SD* 0.0081) (*n* = 3) (*p* = 0.34); zuclopenthixol 0.0143 mg/l (SD 0.0167) (*n* = 9) and 0.0160 mg/l (*SD* 0.0227) (*n* = 2) (*p* = 0.91). In the dose adjustment and control group there was a linear incremental relationship between dose and plasma level (data not shown).

Separate analyses were performed with the exclusion of the 12 patients with a PM profile who were selected due to inhibiting medication (data not shown); however, this exclusion had no effect on the results. There were no differences in outcomes between males and females (data not shown).

### Effect of dose adjustment

No significant effect of dose adjustment was found on psychiatric symptoms, quality of life, or global functioning. Of the 41 patients receiving dose adjustment, six returned to the original dose of the antipsychotics because of deterioration after dose adjustment. Deterioration of psychiatric symptoms resulted in two clinical admissions of outpatients of the dose adjustment group, whereas no admissions were reported in the control group; this difference was not significant.

There was no significant difference in deterioration in psychiatric symptoms between the two groups. In the dose adjustment group, 16 patients (39%) showed a decline in one of three aspects (defining deterioration) compared with 14 patients (34%) in the control group. In patients who deteriorated, the mean prescribed dose of antipsychotics (in DDD) after dose adjustment was equivalent to the DDD in patients who remained stable (data not shown). Table 1 shows the mean changes in scores after dose adjustment (T1-T0). There were no differences in outcomes between males and females (data not shown). Table 2 shows the individual therapeutic plasma levels of antipsychotics (mg/l) metabolized by CYP2D6 of the nine patients in the dose adjustment group who participated in the measurements at T0 and T1.

**Table 2:** Therapeutic plasma levels of antipsychotics (mg/l) metabolized by CYP2D6 of the nine patients in the dose adjustment group measured at T0 and T1.

	**Dose adjustment (%)**	**Plasma level change (%)**	**T0**			**T1**			**T1–T0**		
			**H**	**R**	**Z**	**H**	**R**	**Z**	**H**	**R**	**Z**
Participant 1	−25				0.0120			Undetectable^*^			
Participant 2	−25	−54		0.0130			0.0060			−0.0070	
Participant 3	−25	−44			0.0250			0.0140			−0.0110
Participant 4	−25				Undetectable^*^			0.0050			
Participant 5	STOP			0.0350			Undetectable^*^				
Participant 6	STOP	−65		0.0260			0.0090			−0.0170	
Participant 7	STOP	0	0.0010			0.0010			0.0000		
Participant 8	STOP				Undetectable^*^			Undetectable^*^			
Participant 9	STOP				Undetectable^*^			0.0110			

* *All patients were using depot medication and medication adherence was guaranteed, therefore a lab result of 0 was interpreted as undetectable. H, Haloperidol; R, Risperidone; Z, Zuclopenthixol; STOP, stopped with inhibiting medication*.

### Effect of dose adjustment on side-effects and well-being

Dose adjustment did not result in a significant improvement of parkinsonism, dyskinesia, dystonia, or akathisia. There was a slight improvement (6%) in well-being as measured by the SWN-20. However, this is not considered a clinical relevant finding and is not significant after Bonferroni correction.

### Effect of dose adjustment on metabolic parameters

Table 1 shows changes in metabolic parameters from baseline until after dose adjustment; no significant differences were found between the two groups.

## Discussion

This study rejected the hypothesis that patients with SMI on antipsychotic treatment in a clinical setting benefit from dose adjustment based on the *CYP2D6* genotype or phenotype. Importantly, at baseline, no differences were found in the severity of side-effects or global/psychiatric functioning between the dose adjustment group (with PM, IM, and UM) and control group (with EM). There was no effect of dose adjustment on these parameters.

We expected before we started the study that during years of treatment, clinicians would have optimized dosages to the geno-/phenotype based on side-effects or effectiveness of the drugs used. However at baseline, the CYP2D6 PMs, IMs, and UMs used the same amount of antipsychotics as the EMs. This finding motivated us to find out if dose adjustment could improve the clinical picture. A possible explanation for the absence of an effect, which could also explain the deterioration of some patients, is that long-term use of antipsychotics induces structural brain changes and the brain adapts to the changed dopamine levels (35). It is suggested that antipsychotics play a role in the progressive reduction of brain size and enlargement of ventricular spaces in patients with schizophrenia, which is associated with involuntary movement disorders (36, 37). Studies show that patients with long-term antipsychotic treatment have a threefold increase in loss of dopamine terminals in the substantia nigra (15% per decade vs. 5% in healthy controls) which is suggested to play a role in persistent parkinsonism and tardive dyskinesia (38). Additionally, it is reported that only 3% of patients discontinuing movement disorder-causing agents, resolved spontaneously from tardive syndromes and a reduction of the dosage of antipsychotics did not decrease the severity of parkinsonism (39, 40). It could be that in this clinical population, a dose adjustment to CYP2D6 might have had an effect in an earlier disease stadium but after years of treatment has come too late.

Another possible explanation for these findings is that in both our study groups, the baseline dosage of antipsychotics may have been so high (average DDD 1.65) that D2 receptor occupancy exceeded the optimal window for subjective well-being and to forestall extrapyramidal side-effects (41, 42). This could explain why we found no differences in the prevalence of movement disorders and subjective well-being between the dose adjustment group and control group. However, no improvements in extrapyramidal and psychiatric symptoms were found in our patients using lower dosages of antipsychotics (DDD 1.0, after reduction DDD 0.5).

Lastly, the role of the *CYP2D6* genotype as a major factor in the metabolization of antipsychotics might be overestimated. The present study supports this hypothesis by showing no differences in plasma levels of drugs in the different phenotypes. Another clinical study showed, that a proportion of healthy individuals with a PM genotype are phenotypically EMs as measured by CEIBA metabolization (43).

In clinical practice, in patients with SMI, common factors as co-morbidities, inflammation, age, smoking, and drug/alcohol use, could cause conversion of genotypic PMs into phenotypic EMs and the other way around (44–49). This undetected phenomenon, named phenoconversion, might explain the negative outcome effects in the present study.

Although a small group of patients (*n* = 14) remained stable with lower dosages of antipsychotics, no patients improved in clinical symptoms. This relatively minor saving in direct costs, did not weigh up to the costs of genotyping a large group of patients (*n* = 269).

### Strengths and limitations

This is the first study which prospectively investigated the clinical utility of dose adjustment to the *CYP2D6* genotype or phenotype in patients on antipsychotic medication (20). The representativity was high as we were able to approach all psychiatric patients, with a homogenous African-Caribbean background in a restricted area, i.e., we included all three psychiatric institutes on the island of Curaçao. When including patients, no selection was made regarding the type of psychiatric care, medication, presence of side-effects or treatment response. This has resulted in a heterogeneous group of patients, representative for a general clinical population. It allowed us to analyse the effects of genotyping and dose adjustment in a clinical setting and has led to results with practical clinical value. We have no treatment history of the patients more than 2.5 years before the start of the dose adjustment but we know from clinical practice that patients with SMI make several switches in antipsychotics during the course of their illness and treatment. Earlier studies, in a larger population from this same clinic (40, 50) show this is also true for this population.

Although a large cohort of 269 psychiatric patients was genotyped, only a small number of patients (45) had an extreme geno-/phenotype and used medication metabolized by CYP2D6; therefore, a randomized controlled trial design was not possible. A control group was used to investigate differences between normal and extreme metabolizers before dose adjustment and to monitor possible effects from time. Furthermore, the design of the study, with a rater who was blinded to whether a patient was in the dose adjustment group or the control group, reduced possible expectation bias.

Ideally we would have analyzed the effect of dose adjustment in patients using only one type of antipsychotic medication, due to the small numbers of extreme metabolizers this was not possible. Because all the investigated antipsychotics are metabolized by CYP2D6 as reported in the Flockhart table, it is very unlikely that this accounted for the absence of an effect.

At last, the Food and Drugs Administration provides a list of strong and weak inhibitors and, by inclusion of patients with a PM/IM phenotype based on interacting medication, greatly improved the prediction of the correct phenotype and has increased the power of the study (23, 24). *Post-hoc* power analysis showed that the number of included patients was high enough to demonstrate clinical relevant results. Moreover, not one out of 45 patients showed an evident improvement in side-effects, psychiatric symptoms or functioning after dose adjustment and six patients returned to their original doses, due to deterioration of psychiatric symptoms. This supports the conclusion that adjustment of the dose based on the *CYP2D6* geno-/phenotype had no effect.

## Data availability statement

Datasets are available on request: The raw data supporting the conclusions of this manuscript will be made available by the authors, without undue reservation, to any qualified researcher.

## Author contributions

AK, DV, IP, PG, RvS, PvH, and HH study conception and design. AK, DV, and PG acquisition of data. AK and DV analysis and interpretation of data. AK drafting of manuscript. AK, DV, IP, PG, RvS, PvH, and HH critical revision.

### Conflict of interest statement

The authors declare that the research was conducted in the absence of any commercial or financial relationships that could be construed as a potential conflict of interest. The reviewer AS and handling editor declared their shared affiliation at time of review.

## Footnotes

^1^Flockhart DA. P450 Drug Interaction Table (Division of Clinical Pharmacology, Indiana University). July 12, 2013. Available at: http://medicine.iupui.edu/clinpharm/ddis/main-table/ (Accessed 2013).

^2^Central Bureau of Statistics Curaçao. Available at: http://www.cbs.cw. Accessed 2017.

^3^Switching antipsychotics available at: http://wiki.psychiatrienet.nl/index.php/SwitchAntipsychotics. (Accessed 2014).
